# A missed opportunity to improve practice around the use of restraints and consent in residential aged care: Limitations of the Quality of Care Amendment (Minimising the Use of Restraints) Principles 2019

**DOI:** 10.1111/ajag.12757

**Published:** 2019-12-05

**Authors:** Carmelle Peisah, Tiffany Jessop, Juanita Breen

**Affiliations:** ^1^ Capacity Australia Sydney New South Wales Australia; ^2^ School of Psychiatry Faculty of Medicine University of New South Wales (UNSW) Sydney New South Wales Australia; ^3^ Discipline of Psychiatry Sydney University Medical School Sydney New South Wales Australia; ^4^ Dementia Centre for Research Collaboration UNSW Sydney New South Wales Australia; ^5^ Wicking Dementia Research and Education Centre College of Health and Medicine University of Tasmania Hobart Tasmania Australia

**Keywords:** dementia, law, residential care, restraints

## Abstract

**Objective:**

To explore the meaning and potential role of new Quality of Care Amendment (Minimising the Use of Restraints) Principles 2019, (Principles) which amend Quality of Care *Principles 2014* in improving practice around physical and chemical restraint.

**Methods:**

We examined both Principles and accompanying Explanatory Statement in light of best practices around consent and use of chemical and physical restraint.

**Results:**

The chemical restraint definition is problematic by exclusion of medications for treating mental disorders, physical illness or physical conditions, which is not considered restraint. Inexplicably, physical restraint requirements are more rigorous than chemical restraint requirements, where assessment is optional, and consent sometimes obtained, after use, and from the person's “representative,” rather than the person first, followed by their proxy decision‐maker.

**Conclusions:**

Although a start in promoting best practice around physical restraint, the Principles do not address the status quo of poor practice around chemical restraint and may instead codify it.

**Practice Impact**

Clinicians need to be mindful that improved practice around chemical restraint and consent beyond the new Quality of Care Amendment (Minimising the Use of Restraints) Principles 2019 legislation is urgently needed.

## 1 INTRODUCTION

In September 2018, in the wake of extensive media coverage and sanctions placed on a significant number of residential aged care facilities (RACFs), the Hon. Ken Wyatt, the Minister for Senior Australians and Aged Care, announced a Royal Commission into Aged Care Quality and Safety. The Royal Commission commenced on 11 February 2019 and has had, to date, a strong focus on both overuse and inappropriate use of physical and chemical restraint and the lack of consent for the use of restraint in aged care. Minister Wyatt was outspoken about his desire to tackle these issues and on 2 April 2019 made the Quality of Care Amendment (Minimising the Use of Restraints) Principles 2019. Section 96‐1 of the *Aged Care Act 1997* (Aged Care Act) provides that the Minister may, by legislative instrument, make Quality of Care Principles, providing for matters required or permitted by Part 4.1 of the Act. The *Quality of Care Amendment (Minimising the Use of Restraints) Principles 2019* (Amending Principles) amends the Quality of Care Principles 2014 (Quality of Care Principles) to limit the use of chemical and physical restraint by approved providers of residential care and short‐term restorative care in a residential setting (https://www.legislation.gov.au/Details/F2019L00511). The previous Quality of Care Principles 2014 referred to neither chemical restraint nor consent.

The overuse and misuse of both chemical and physical restraint may constitute elder abuse^1^ and has long been identified as problematic,^2^, ^3^ and in some ways getting worse where psychotropic prescribing is concerned.^4^, ^5^ The use of restraints has been driven by a need to “manage” disturbed perception, thinking, mood and behaviour arising in the context of dementia, also known as behavioural and psychological symptoms of dementia (BPSD) or changed behaviours.^6^, ^7^ BPSD include aggression, agitation, wandering, anxiety and depression and are often an expression of unmet needs such as pain, loneliness or need for intimacy, hunger, boredom and overstimulation.^3^ As such, international consensus best practice guidelines recommend the use of multidisciplinary, individualised, psychosocial approaches as the first‐line approach to BPSD. Such person‐centred care relies on observation, measurement and monitoring of BPSD to assess the antecedents, triggers and consequences of behaviours.^3^, ^8^, ^9^, ^10^, ^11^ Only after these person‐centred non‐pharmacological approaches have been trialled and failed, or there are risks to the safety of the person or those around them, should any type of restraint be used.^3^ For these reasons, and human rights concerns,^12^ restraint use should be avoided where possible.

Despite these guidelines, more often than not there is recourse to chemical or physical restraint,^2^, ^3^, ^4^, ^5^ usually without consent from the person or their proxy decision‐maker.^13^ Under Common Law, no treatment can be undertaken without consent of the person if they are a competent adult (ie a person with capacity to make the decision).^14^ For valid consent, the person must be: (a) competent (have capacity) to make the treatment decision (in this case, restraint); (b) acting voluntarily without pressure or duress; and (c) provided with enough relevant information about the treatment options, alternatives and material risks, presented in a form that can be understood to enable the person to make the decision.^14^ There is a presumption of capacity for all adults, regardless of their diagnosis or whether they live in a nursing home, and a valid “trigger” must exist to rebut this presumption and prompt an assessment. If a practitioner assesses a person as lacking capacity to give consent to treatment, they must seek consent from a proxy decision‐maker, a process governed by various legislative regimes across Australia.^14^

Lack of consent has long been recognised as a problem internationally. In 1990, a study of Massachusetts nursing homes showed that informed consent was not considered an issue and decision‐making capacity was not tested. The usual practice was for capacity to be presumed until a patient failed to acquiesce to treatment, and only at that point would the issue of capacity be fully addressed.^15^ Despite policies and guidelines mandating consent, and a range of international initiatives to educate clinicians about consent,^14^, ^16^ proper consent is obtained in only about 6.5% of cases.^17^ In the Australian HALT study of 140 RACF residents taking antipsychotic medications for changed behaviours across 23 RACFs, only one case met the NSW legal requirement of written proxy consent. Another 20 participants had a file note recording a conversation between a clinician and a family member, leaving 84% of participants with no evidence of a consent process.^13^

The issues surrounding the use of restraint are many and complex. This paper aims to explore the meaning and potential role of the new Quality of Care Amendment (Minimising the Use of Restraints) Principles 2019, hereafter known as “The Principles” in improving practice around physical and chemical restraint, and its limitations in doing so.

## 2 METHODS

We examined both the Principles and their accompanying Explanatory Statement in light of international consensus best practice guidelines around both consent and the use of psychotropics. The Principles provide definitions of chemical and physical restraint under Schedule 1. Conditions for the use of chemical and physical restraint are also provided under Part 4A—Minimising the use of Physical and Chemical Restraint, including the steps Providers must take before using such restraints.

## 3 RESULTS

### 3.1 Definitions

It is noted that under Schedule 1—Amendments, 1. Section 4:

***chemical restraint*** *means a restraint that is, or that involves, the use of medication or a chemical substance for the purpose of influencing a person’s behaviour, other than medication prescribed for the treatment of, or to enable treatment of, a diagnosed mental disorder, a physical illness or a physical condition.*

***physical restraint*** *means any restraint other than:*

*(a)* *a chemical restraint; or*

*(b) the use of medication prescribed for the treatment of, or to enable treatment of, a diagnosed mental disorder, a physical illness or a physical condition.*

***restraint*** *means any practice, device or action that interferes with a consumer’s ability to make a decision or restricts a consumer’s free movement.*

Specifically, with regard to chemical restraint, the Explanatory Statement expands on this, stating: “it is not chemical restraint if those medications are used to treat a diagnosed mental disorder” (eg antipsychotics to treat psychosis associated with disorders such as schizophrenia or bipolar disorder).

This definition of chemical restraint in the legislation is problematic by the exclusion of “mental disorder, a physical illness or a physical condition.” Read in isolation, without the caveat in the Explanatory Statement that antipsychotics used to treat psychosis in mental disorder is not chemical restraint, there is a risk that this will be interpreted as an “opt‐out clause” for best practice minimisation of chemical restraint and the obtaining of consent in these circumstances. Specifically, antipsychotics used to influence behaviour per se in serious mental disorders still constitute restraint. Prescribing these medications also still requires consent. Older people with mental illness in residential care—whose needs are complex, yet often unmet^18^, ^19^—equally deserve best practice approaches from aged care providers. This includes minimisation of chemical restraint, using person‐centred assessment and careful consideration of the reasons for, and alternatives to restraint. A practical example is the older person with schizophrenia, who is sexually disinhibited or aggressive because of unmet intimacy needs or loneliness. Using antipsychotics or other psychotropics to treat this is still chemical restraint regardless of whether the person has schizophrenia.

Other potential misinterpretations of these exclusion criteria for chemical restraint include delirium caused by a physical illness or physical condition, treatment recommendations for which no longer include psychotropics.^20^ Thus, although the legislation suggests otherwise, treating behavioural disturbance in delirium still constitutes chemical restraint. Finally, many clinicians would argue that dementia itself is very much a “physical condition” as it involves neurodegenerative disease, thereby potentially excluding the treatment of behaviour disturbance in dementia from the definition of chemical restraint.

Exceptions to what is covered by the definition of chemical restraint are too wide, potentially rendering the legislation ineffective in reducing its misuse or overuse.

### 3.2 Conditions for use of chemical and physical restraint

Under Section 15F (1), it is noted that an approved provider must not use a physical restraint unless:

- An Approved Health Practitioner Who Has Day‐To‐Day Knowledge Of The Consumer Has:
  - Assessed The Consumer As Posing A Risk Of Harm To The Consumer Or Any Other Person, And As Requiring The Restraint; And
  - Documented The Assessment, Unless The Use Of The Restraint Is Necessary In An Emergency; And
- Alternatives To Restraint Have Been Used For The Consumer To The Extent Possible; And
- The Alternatives To Restraint That Have Been Considered Or Used Have Been Documented, Unless The Use Of The Restraint Is Necessary In An Emergency.

Notably, in stark contrast, under Section 15G(1), a provider must not use chemical restraint unless:

- A medical practitioner or nurse practitioner has assessed the consumer as requiring the restraint and has prescribed the medication the use of which is, or is involved in, the restraint; and
- The practitioner's decision to use the restraint has been recorded in the care and services plan documented for the consumer in accordance with the Aged Care Quality Standards set out in Schedule 2.

Under 15G(2) b, having decided to use restraint (ie after using restraint) the provider must ensure that the care and services plan identifies:

- The Consumer's Behaviours That Are Relevant To The Need For The Restraint;
- The Alternatives To Restraint That Have Been Used (If Any);
- The Reasons The Restraint Is Necessary (If Known By The Approved Provider);
- The Information (If Any) Provided To The Practitioner That Informed The Decision To Prescribe The Medication.

Such requirements for chemical restraint suggest merely documentation of alternatives to restraints (“if any” have been used) after use.

### 3.3 Consent requirements

Under Section 15F (e) with regard to physical restraint, the provider must have the informed consent of the consumer or their representative to the use of the restraint, with no such requirement mandated for chemical restraint under 15G, only “if it is practicable to do so” or “after the restraint starts to be used.” Obtaining consent after treatment is not consent. Moreover, the requirement to inform the “consumer's representative” is contrary to the law and best practice, which stipulates that an attempt should always be made to obtain consent from the person themselves first, followed by a proxy decision‐maker if necessary.^3^, ^14^ The current requirement presumes that all people requiring chemical restraint lack capacity to give consent, contrary to the presumption of capacity outlined above.

## 4 CONCLUSIONS

The Principles are a start in promoting best practice with regard to restraints, particularly physical restraints, as are flow‐on initiatives such as the *Self‐assessment tool for recording consumers receiving psychotropic medications* 21 and our own work with WebsterCare.^22^ However, the less rigorous requirements for chemical restraint, with optional assessment, and consent sometimes obtained, after restraint use, and from the person's “representative,” rather than the person themselves first followed by their proxy decision‐maker, do not address the status quo of poor practice and may serve to codify it. Restraint is restraint, whether physical or chemical. To make one more permissible, or easier to obtain, endorses its use and sends a strong message that it is a preferable option for managing changed behaviours. The key message should not be which type of restraint is preferable, but rather to, as the legislation purports, minimise restraint.

“Quality of Care” with regard to the use of chemical restraint means restraint used as last resort with stringent safeguards such as mandatory consent and in least restrictive form (ie for chemical restraint, minimal dose, short duration, avoiding polypharmacy), and monitoring for effect and harm.^23^ Clearly, other strategies for improving practice around chemical restraint and consent are required, beyond this legislation.
